# Modeling the distribution of the endemic Turkish moss species *Cinclidotus bistratosus* Kürschner & Lüb.-Nestle (Pottiaceae) under various climate change scenarios

**DOI:** 10.3389/fpls.2025.1659115

**Published:** 2025-09-15

**Authors:** Gökhan Abay, Serkan Gül

**Affiliations:** ^1^ Department of Landscape Architecture, Faculty of Engineering and Architecture, Recep Tayyip Erdogan University, Rize, Türkiye; ^2^ Department of Biology, Faculty of Arts and Sciences, Recep Tayyip Erdogan University, Rize, Türkiye

**Keywords:** biomod2, climate, endemic moss species, conservation strategies, global warming, species distribution modeling

## Abstract

The extant literature on the subject is inconclusive, with only a paucity of studies addressing variations in the distribution patterns of moss species, particularly those with restricted distributions, in the framework of climate change. Consequently, we constructed simulated current and predicted prospective potential distribution models of *Cinclidotus bistratosus*, a narrow-range endemic moss species belonging to Türkiye, using the CMCC-ESM2, HadGem3-GC31-LL, and MIROC6 climate models. The purpose of this paper is to examine the distinct habitat requirements of the endemic moss, the key environmental factors that influence its distribution, and the distribution changes of the species under climate change over a substantial spatial-temporal scale (between the periods 2021-2100). Precipitation of driest, hottest and coldest quarters has been identified as a key factor influencing *C. bistratosus* distribution models. The findings of this study indicate that the highest probability of habitat suitability for *C. bistratosus* is currently in the coastal regions of western and southern Türkiye. However, future projections indicate a substantial decline in suitable habitats and a potential expansion towards northern regions of the country. In the scenario of prospective climate warming, the appropriate habitat of *C. bistratosus* may shift towards northern and high-altitude regions under the SSP5-8.5 climate scenario. However, the species will not entirely withdrawal from the Mediterranean distribution range, and its possible distribution will be restricted in Türkiye. The present study provides significant information and support for understanding the effects of climate change on the distribution of *C. bistratosus*, as well as its future distribution and conservation strategies.

## 1 Introduction

The scientific community now acknowledges climate change to be among the most pressing issues presently posing a threat to both the natural world and biodiversity (He et al., 2016). Especially in the last 30-40 years, this phenomenon has become a topic of great interest to society. This interest is directly related to the phenomenon of global warming, which is caused by an increase in concentrations of greenhouse gases within the atmosphere (Gignac, 2001). Since the pre-industrial era, industrialization and anthropogenic greenhouse gas emissions have contributed to the increase in global warming (Zanatta et al., 2020). Industrial carbon dioxide (CO_2_) emissions, one of the greenhouse gases, have led to a rapid increase in atmospheric CO_2_ levels, causing global warming and consequently climate change (Mohanasundaram and Pandey, 2022). One sign of global climate change is that there has been a one degree rise in the earth’s temperature over the last century (Field et al., 2014; He et al., 2016). It is expected that the average global temperature will increase by 1.5°C between 2030 and 2052. This value is projected to be two to three times higher in Arctic regions (Zanatta et al., 2020). Predicted short-term (<100 years) increases in global temperature will result in increased concentrations of greenhouse gases, which will significantly impact the atmosphere, particularly in vegetation zones at mid- and high latitudes. It is clear from past climate changes that ecosystems and species will be affected (Gignac, 2001). Unfavourable climatic conditions can have an impact on many plants, including both vascular and non-vascular plants such as bryophytes including mosses (Bryophyta), liverworts (Marchantiophyta), and hornworts (Anthocerotophyta). While some plants may expand their distributional range due to an increase in suitable conditions, others may experience changes or a reduction in favourable conditions (Číhal, 2023; Ferretto et al., 2023).

In the last two to three decades, the potential impacts of climate change on plant biodiversity have been more intensively investigated. Although many studies have biased mainly on seed plants (Thuiller et al., 2005; Walck et al., 2011; Inouye, 2020; Xiong et al., 2022; Hernandez et al., 2023; Janni et al., 2024), there are also studies on small-structured plants of the ecosystems such as bryophytes (Bates and Preston, 2011; Sérgio et al., 2011; Ferreira et al., 2016; Patiño et al., 2016), and lichens (Singh et al., 2018; Mallen-Cooper et al., 2023; Stanton et al., 2023).

Bryophytes are a group of early land plants that produce spores. It is evident that they possess specific ecophysiological and biological characteristics that render them optimal subjects for the investigation of the repercussions of climate change (Tuba et al., 2011; Patiño et al., 2016). They grow in almost all terrestrial and freshwater environments and have a unique physiology and ecology that sets them apart from vascular plants. This means they differ in their ability to influence elemental, energy and water cycles. Poikilohydric condition means that their water content is directly regulated by environmental humidity, the ability to tolerate desiccation, along with poorly developed conduction systems and the lack of gametophyte stomata and cuticles, make bryophytes more sensitive to atmospheric chemical deposition compared to vascular plants (Turetsky, 2003; Patiño et al., 2016). Despite this, they play important roles in both terrestrial (Fenton et al., 2015) and aquatic ecosystems (Stream Bryophyte Group, 1999), regulating the global carbon cycle, particularly in arctic ecosystems (Douma et al., 2007). Bryophytes are of significant importance with regard to the maintenance of the water balance and the process of humus formation. They are able to fix nitrogen, act as pioneer colonizers in succession, indicate pollution and heavy metal presence, and serve as site indicators (Bahuguna et al., 2013). Additionally, they can help control soil erosion and provide habitats for microorganisms. In conclusion, bryophytes are a crucial component of biodiversity and play important roles in ecosystem dynamics. They also contribute to mitigating global warming issues (Chimyang et al., 2022).

The Turkish bryophyte flora currently with 1244 taxa (215 liverworts, 1025 mosses, four hornworts) was reported in the study of identification keys of Turkish bryoflora performed by Kürschner and Erdağ (2023). According to the list of endemic bryophyte species in Türkiye (formerly, Turkey) (Erdağ and Kürschner, 2017), the number of which may vary according to taxonomic and floristic progress, a total of 10 taxa (seven mosses and three liverworts) are endemic to Türkiye, which is ca. 0.8% of the Turkish bryoflora. Bryophytes are characterized by extremely low levels of endemism in Turkish floras. The endemism of mosses and liverworts from Türkiye revealed ca. 0.6% and ca. 0.2%, respectively, in the whole bryoflora. The endemism rate of mosses in Türkiye is ca. 0.7% within their own division (Bryophyta). In the liverworts (division Marchantiophyta), this ratio is ca. 1.4%. There is no endemic hornwort species belonging to the division Anthocerotophyta in Türkiye.

The objective of this study was to project the potential impact of climate change on the distribution of the endemic moss species *Cinclidotus bistratosus* Kürschner & Lüb.-Nestle in Türkiye and to investigate whether species distribution modelling (SDM) estimates would change with the inclusion of projected changes in this moss species habitats (Ferretto et al., 2023). In particular, the following questions are addressed in this study: (i) to what extent will the species’ distributions and elevational ranges change under different scenarios of climate change? (ii) the question of whether this endemic moss will be equally affected throughout its distribution range is one that has yet to be answered (Patiño et al., 2016), (iii) in conclusion, the prediction of the consequences of prospective multi-scale environmental alterations on the endemic *C. bistratosus* will facilitate the formulation of conservation strategies and the making of informed decisions (Lomba et al., 2010).

## 2 Materials and methods

### 2.1 Studied area

Türkiye is geographically located between approximately 36° – 42° North latitude and 26° – 45° East longitude (Evrendilek et al., 2007). It possesses a rich genetic diversity thanks to its climatic and topographic characteristics with a land surface area of 783,562 km^2^ (İzmirli Güzel and Gül, 2023). The country hosts three distinct biodiversity hotspots: the Caucasus, located in northeastern Türkiye; the Iran-Anatolia hotspot, encompassing a large portion of Central and Eastern Anatolia; and the Mediterranean Basin, which covers the western and southern parts of the Anatolian Peninsula (Myers et al., 2000). Şekercioğlu et al. (2011) also reported four major mountain belts in Türkiye. These are the Yıldız Mountains on the European side of the country; the Taurus Mountains, which lie between the Mediterranean coast and Eastern Anatolia; the Köroğlu and Kaçkar Mountains within the North Anatolian Mountains; and the Anatolian Diagonal, which extends from the northeast to the Mediterranean. Also, the country’s average elevation is around 1,130 metres; more than 25% of its land lies above 1,200 metres, with elevations exceeding 1,500 metres, particularly in the Eastern Anatolia region (Kömüşcü and Aksoy, 2024).

### 2.2 Studied species and species occurrences

Of the 10 endemic bryophyte taxa distributed in Türkiye (Erdağ and Kürschner, 2017), *Cinclidotus bistratosus* was selected as the study material. The reason for this choice is that the number of existence records for the other nine bryophyte taxa is below five. The number of geographical coordinates of the endemic moss *C. bistratosus* available in the literature is only five (Kürschner and Erdağ, 2021). In accordance with the recommendations of Cerrejón et al. (2022), only those with a minimum of five occurrences (≥5 occurrences) were ultimately utilized, given the meaningful predictions that were observed.

*C. bistratosus* is a species of moss that grows in areas with continuous water streams and is characterized by its compact and hygrophytic nature. Its initial description was as a new species from the steep mountain passes of the Taurus Mountains of Southern Anatolia (Köprülü Canyon National Park). The species was collected in the flood zone of the Köprülü River, where it grows on rocks exposed to summer drought and strong insolation as well as inundation (Kürschner and Lübenau-Nestle, 2000). Following the initial documentation of this species in Türkiye, numerous authors have subsequently reported its occurrence in proximate localities. Kırmacı and Özçelik (2010) documented the presence of this species in Türkiye, Antalya, on calcareous rock in the Beşkonak- Başlar neighborhood. Erdağ and Kürschner (2011) collected the moss on in-water rock in Bolhasan bridge locality in Köprülü Canyon National Park and on rocks exposed to inundation in the flood zone of Köprü River towards the end of the steep canyon between Oluk bridge and Çaltepe, 15 kilometres north of Beşkonak in Antalya. Finally, Özçelik et al. (2016) reported on in-water rocks in Beyreli village and on in-water rocks near Dimçayı stream in Alanya district, Antalya.

The occurrence records of *C. bistratosus* in Türkiye were compiled from above mentioned sources (Kürschner and Lübenau-Nestle, 2000; Kırmacı and Özçelik, 2010; Erdağ and Kürschner, 2011; Özçelik et al., 2016; Kürschner and Erdağ, 2021). In the absence of precise geographical coordinates, we employed the Google Earth platform (https://earth.google.com/web/) to undertake toponymic geocoding, as in the work of Cong et al. (2020). The total number of documented occurrences of the species in Türkiye was five.

### 2.3 Environmental variables and climate change scenarios

A total of 19 bioclimatic datasets were retrieved from WorldClim version 2, for the present variables. 1 (available at https://www.worldclim.org/). These datasets span the period 1970–2000, have a spatial resolution of 30 seconds (~1 km²) and are delivered in GeoTiff (. tif) format (Fick and Hijmans, 2017). Data for three global climate models (GCMs) (CMCC-ESM2, HadGEM3-GC31-LL, and MIROC6) were collected for future climate variables, as were projections for two shared socio-economic pathways (SSP 1-2.6 and 5-8.5). This covers the time intervals 2021–2040, 2041–2060, 2061–2080, and 2081–2100 at 30s spatial resolution according to CMIP6 downscaled climate projections. The second-generation CMCC Earth System Model, or CMCC-ESM2, is the first of the GCMs. It has been significantly improved, especially in terms of integrating a variety of marine and terrestrial biogeochemical processes. A greater variety of carbon pools and plant functional kinds are included in this revised edition, expanding its utility. Its accuracy in replicating terrestrial biogeochemistry is further enhanced by its predicted representation of the nitrogen cycle (Lovato et al., 2022). The atmosphere, ocean, sea ice, and land are all represented by linked components in the second model, HadGEM3-GC3.1-LL (Hadley Centre Global Environmental Model), which is set up in a Global linked 3.1 configuration. Through the simulation of interactions between these essential elements, this integrated system improves our understanding of global environmental dynamics (Andrews et al., 2020). Finally, the Center for Climate System Research (CCSR), the forerunner of the Institute of Atmosphere and Ocean Research at the University of Tokyo, collaborated to create the Japanese climate model known as MIROC (Model for Interdisciplinary Research on Climate). The National Institute of Environmental Studies (NIES) and the Japan Agency for Marine-Earth Science and Technology (JAMSTEC) collaborated to create this model. Atmosphere, land, and sea-ice-ocean are the three distinct sub models that make up the most recent version, MIROC6 (Tatebe et al., 2019). The SSP 1-2.6 scenario, which forecasts a significant drop in carbon emissions by 2050, is an optimistic view of the Shared Socioeconomic Pathways (SSPs). Global temperatures are predicted to stabilize at about 1.8°C as a result of this decrease. A more bleak trajectory is shown by the SSP 5-8.5 scenario, in which CO_2_ emissions increase until 2050, resulting in a projected 4.4°C increase in average temperature (Pielke et al., 2022).

Variance Inflation Factor (VIF) (Marquardt, 1970) values were calculated using the *usdm* package (Naimi et al., 2014) under the *sdm* package (Naimi and Araújo, 2016) to reduce highly correlated bioclimatic variables. According to Alemayehu et al. (2024), variables with a threshold value higher than 10 were considered collinear and therefore ignored. This method computes the correlation coefficient after first extracting the bioclimatic parameters from the species’ geographic reference. As a result, the factors that had the greatest impact on the species’ spread were identified. Thus, the precipitation of coldest quarter (bio19), the precipitation of driest quarter (bio17), and the precipitation of warmest quarter (bio18) were selected and used for future analyses.

### 2.4 Species distribution modeling

BIOMOD2 package was utilized for ensemble species distribution modeling (eSDM) (Guéguen et al., 2025). The following four algorithms were used: Random Forest (RF), Generalized Linear Model (GLM), eXtreme Gradient Boosting Training (XGBOOST), and Maximum Entropy (MAXENT). Because absence records were unavailable, 500 pseudo-absence records were produced at random for each model, which is based on presence-absence algorithms (Lobo and Tognelli, 2011; Iturbide et al., 2015; Hamid et al., 2019). For every model, 20% of the data (validation set) was chosen at random for algorithm performance and 80% of the data (training set) was chosen at random for model calibration (Guisan et al., 2017). Within the Biomod2 framework, a specific model configuration involved executing each of the four algorithms three times, resulting in a total of twelve individual runs. To ensure robust model validation, 500 iterations were performed. Subsequent to preliminary evaluations, only modeling approaches demonstrating a Receiver Operating Characteristic (ROC) value exceeding 0.9 were retained for the development of the final ensemble model. Test samples for this process were chosen via the bootstrap method. The performance of the resulting Biomod2 models was assessed using both the Area Under the ROC Curve (AUC) and the True Skill Statistic (TSS). For AUC, values below 0.6 signified failing performance, 0.6 to below 0.7 indicated poor performance, 0.7 to below 0.8 suggested moderate performance, 0.8 to below 0.9 implied good performance, and 0.9 to 1 indicated excellent performance (Amaral et al., 2023). TSS scores, ranging from −1 to 1, defined values above 0.75 as indicative of excellent model performance (Allouche et al., 2006).

## 3 Results

### 3.1 Model evaluation and environmental factors

**Figure 1:**
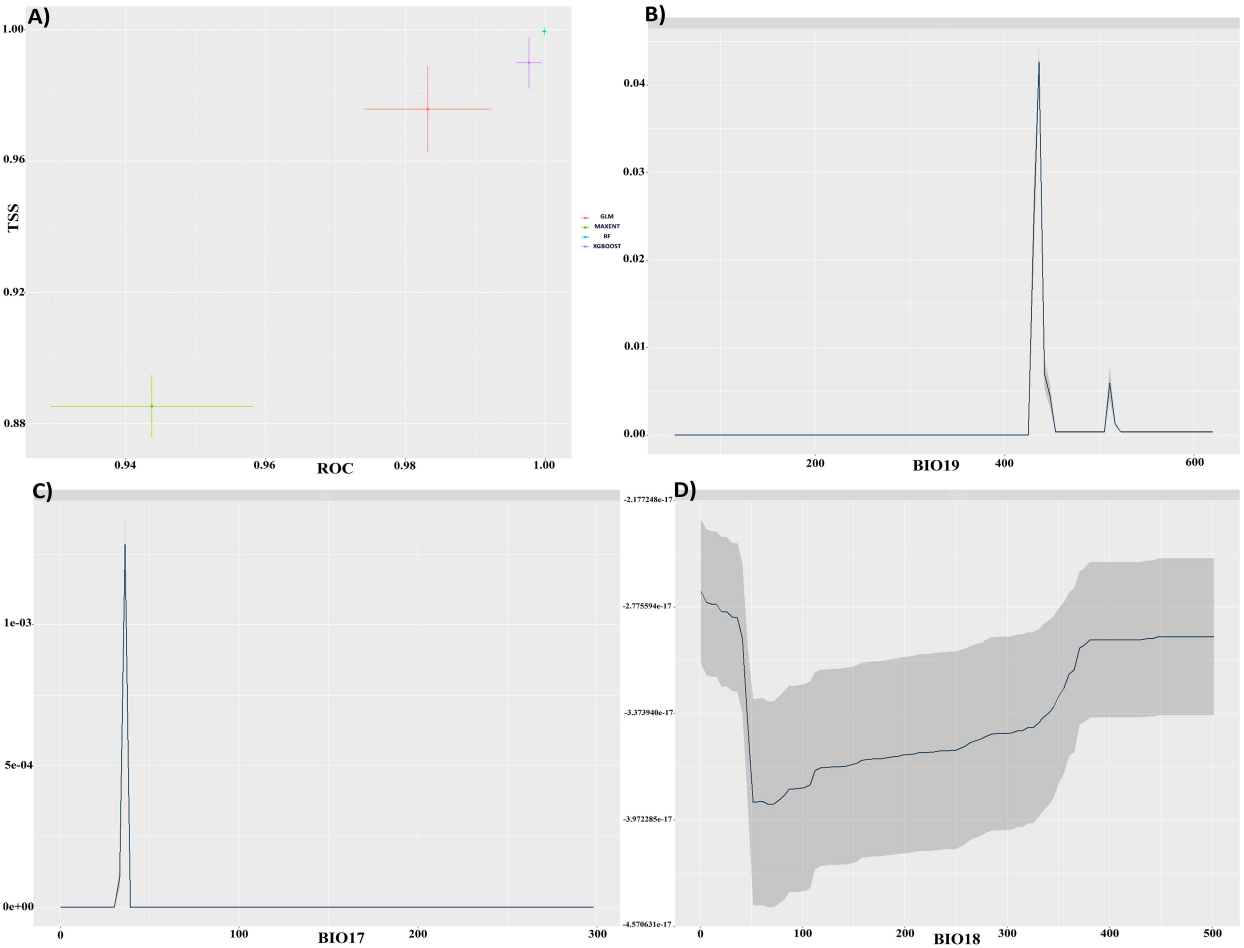
**(A)** TSS and ROC evaluations for four models. Response curves for precipitation of coldest quarter (bio19) **(B)**, Precipitation of driest quarter (bio17) **(C)**, Precipitation of warmest quarter (bio18) **(D)**, respectively.

The predictive accuracy of the RF, GLM, MaxEnt, and XGBOOST models was evaluated. Each model yielded robust results for *C. bistratosus*, with TSS scores exceeding 0.85 and AUC values exceeding 0.94 (**Figure 1A**). An ensemble model (EM) was created by integrating the outputs of all four models and prioritizing the one with the highest performance metrics, as a consequence of these results. The ensemble approach produced superior results for *C. bistratosus*, with TSS values exceeding 0.96 and AUC values exceeding 0.98. Accordingly, the analyses in this study were carried out exclusively on the outputs of this ensemble model. The correlation coefficient showed that three bioclimatic factors were still present among all the environmental variables. Based on the correlation metric, the precipitation of coldest quarter (bio19) had the biggest average effect, at 81.3%. The next highest was the amount of rain that fell in the precipitation of driest quarter (bio17), which was 68.6%. The amount of rain that fell in the precipitation of warmest quarter (bio18) was 36.7% (**Figures 1B–D**).

### 3.2 The present and future projections

**Figure 2:**
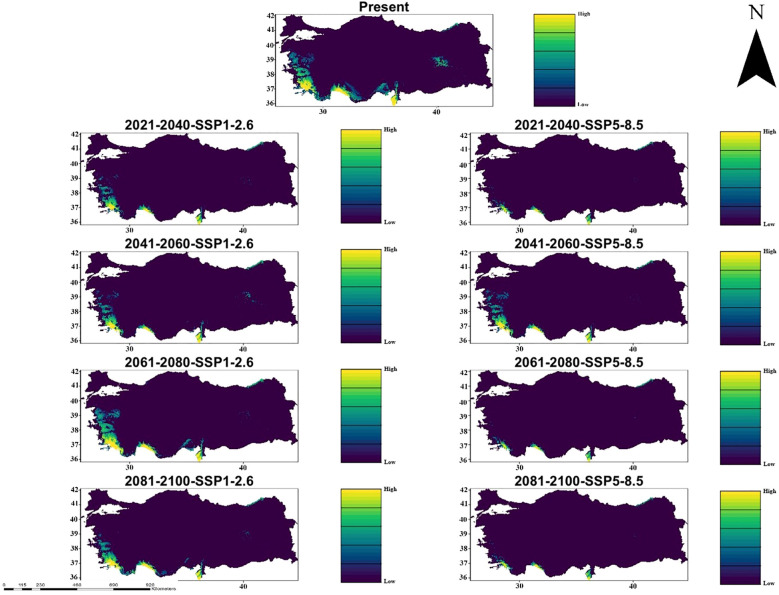
Ensemble species distribution patterns of *C. bistratosus* between present and future based on CMCC-ESM2 climate model.

According to CMCC-ESM2 climate model, species distribution modeling results show that there are records of habitat suitable for *C. bistratosus* under both SSP1-2.6 and SSP5-8.5 climate scenarios in different future periods (2021-2100). Today’s habitat conformity map indicates the highest probability in coastal and lowland regions, especially in the western and southern parts of Türkiye. However, future projections, especially within the scope of SSP5-8.5, reveal that there is a gradual decrease in appropriate habitat. Although some constraints are observed under SSP1-2.6, especially in the inner regions, the appropriate habitat remains relatively constant until 2100. In contrast, SSP5-8.5 results show that there is a more significant decrease in habitat compliance and that the nuclear habitat areas were significantly shrinking between 2081-2100. Southeast and coastal regions exhibit the most important changes with some areas that maintain suitability under SSP1-2.6 but become quite inappropriate below SSP5-8.5 (**Figure 2**).

**Figure 3:**
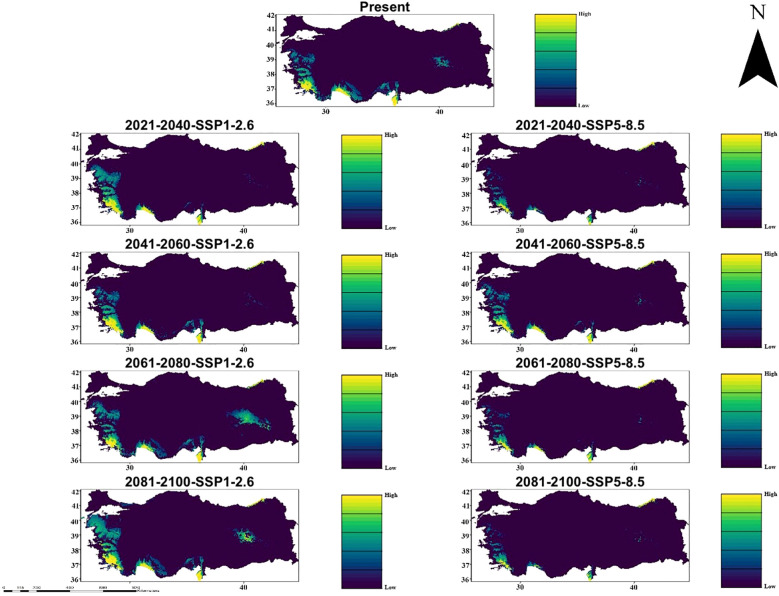
Ensemble species distribution patterns of *C. bistratosus* between present and future based on Hadgem3-GC31-LL climate model.

In the Hadgem3-GC31-LL climate model, significant changes in the suitability of habitat for *C. bistratosus* have emerged under both SSP1-2.6 and SSP5-8.5 scenarios. The present distribution shows that appropriate habitats are concentrated in the coastal and lowland regions and Türkiye has high suitability in the western, southern and northeastern parts. However, future projections show that there are significant decreases in appropriate habitat, especially within the scope of SSP5-8.5. Although there is a gradual decrease in the inner regions within the scope of SSP1-2.6, the suitability of habitat remains relatively constant, and the suitability maintains mainly along the western and southern coasts. On the other hand, within the scope of SSP5-8.5, the suitability of habitat is particularly contracted, especially in the middle and inner regions, and only a few coastal shelters remain at 2081-2100 (**Figure 3**).

**Figure 4:**
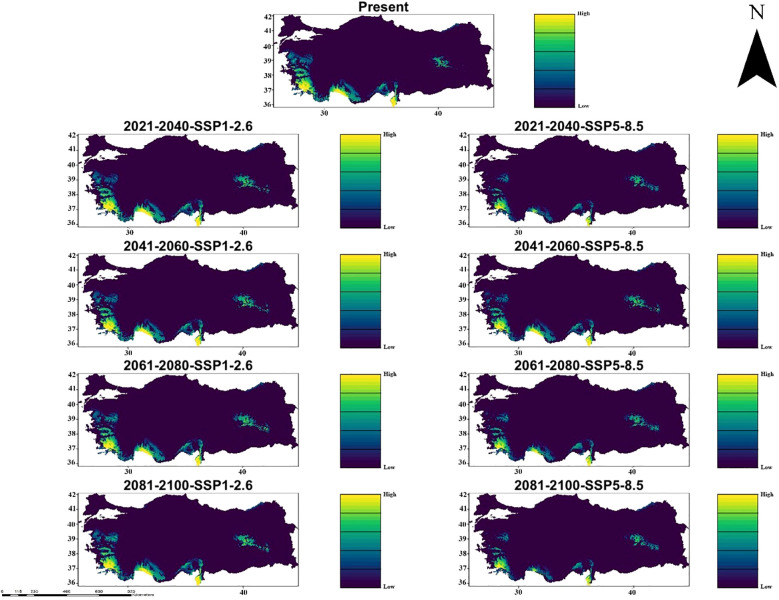
Ensemble species distribution patterns of *C. bistratosus* between present and future based on MIROC6 climate model.

Similarly, in the MIROC6 climate model, under the climate scenarios from 2021 to 2100 (SSP1-2.6 and SSP5-8.5), it indicates significant changes in the suitability of habitat for *C. bistratosus*. Within the scope of SSP1-2.6, although there is a decrease in compliance in some inner regions, the suitable habitat remains relatively constant until 2100. Coastal zones continue to support suitable habitats, especially on the west and southern coast. On the other hand, within the scope of SSP5-8.5, the habitat suitability decreases more harshly, the internal regions become largely unsuitable and only partly part of the coastal regions have suitable habitats. Between 2081-2100, within the scope of SSP5-8.5, the suitable habitat of the species is primarily limited to small shelters along the west and southern coasts (**Figure 4**).

### 3.3 Future changes in habitats of *C. bistratosus*

**Figure 5:**
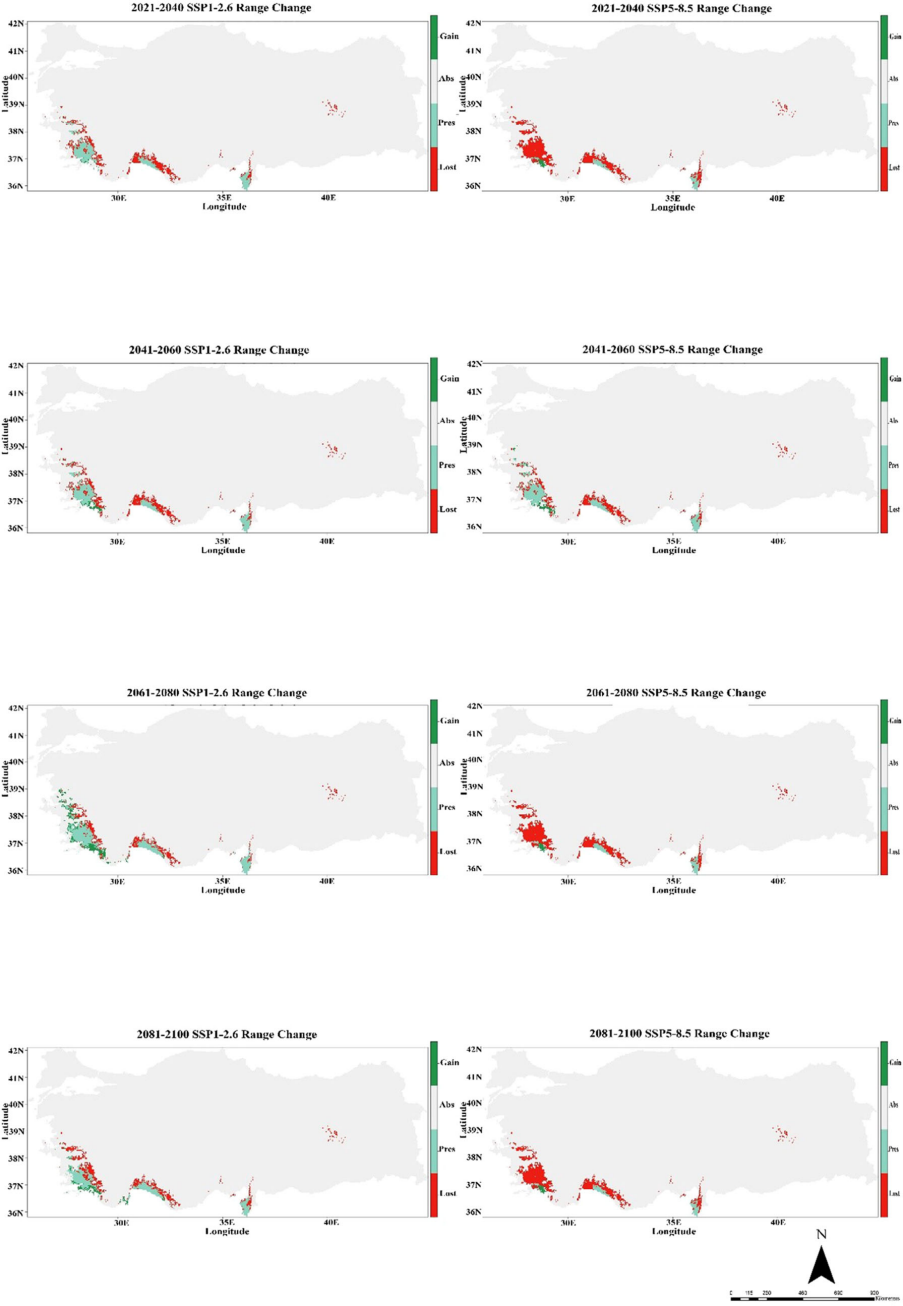
Habitat change patterns of *C. bistratosus* between present and future based on CMCC-ESM2 climate model. Gain: habitat that becomes suitable in the future. Stable (Pres): habitat that is suitable now and will remain suitable in the future, Loss: areas that are in the current distribution but will become unsuitable in the future, Abs: areas that are unsuitable both in the present and in the future.

**Figure 6:**
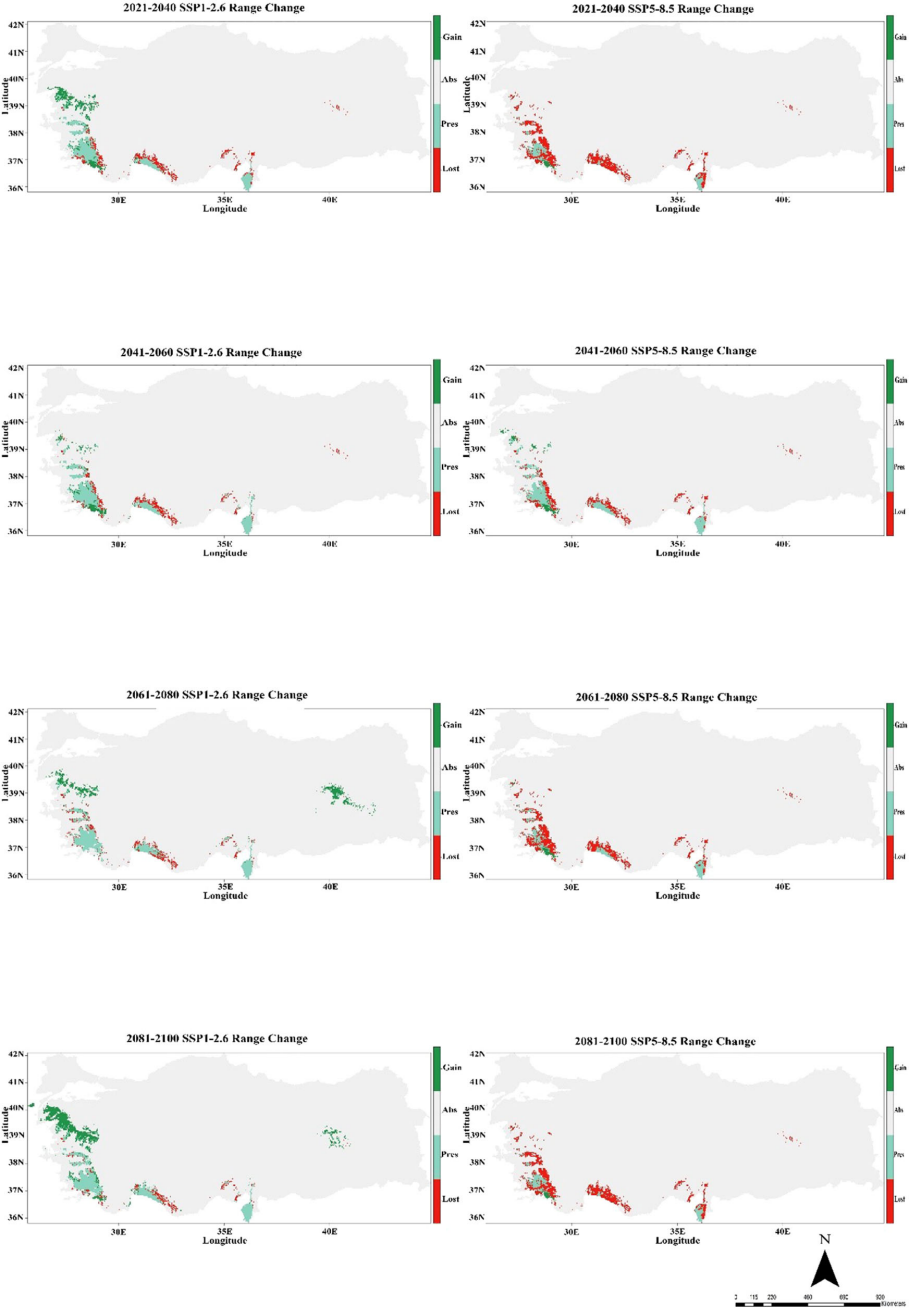
Habitat change patterns of *C. bistratosus* between present and future based on Hadgem3-GC31-LL climate model. Gain: habitat that becomes suitable in the future. Stable (Pres): habitat that is suitable now and will remain suitable in the future, Loss: areas that are in the current distribution but will become unsuitable in the future, Abs: areas that are unsuitable both in the present and in the future.

**Figure 7:**
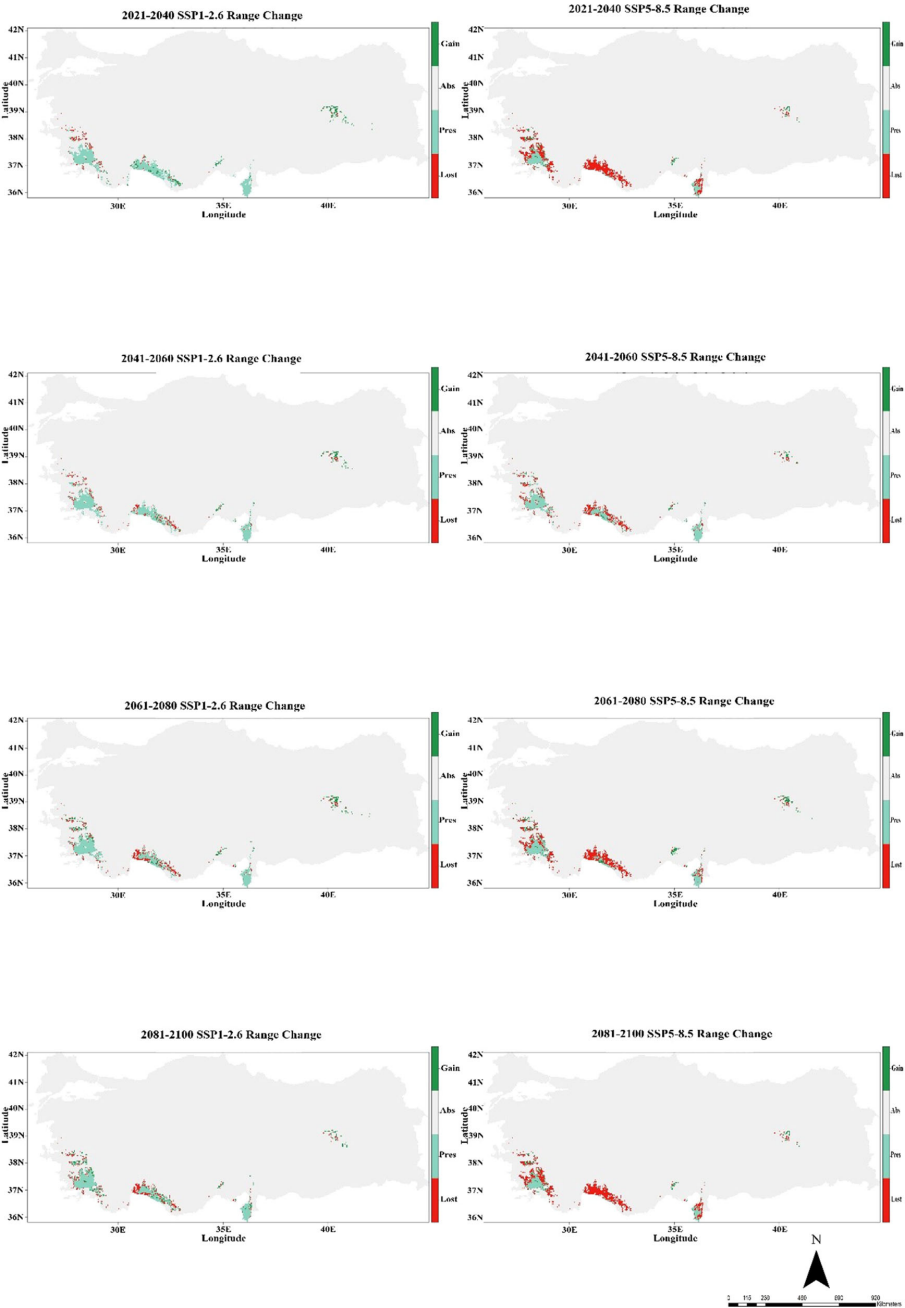
Habitat change patterns of *C. bistratosus* between present and future based on MIROC6 climate model. Gain: habitat that becomes suitable in the future. Stable (Pres): habitat that is suitable now and will remain suitable in the future, Loss: areas that are in the current distribution but will become unsuitable in the future, Abs: areas that are unsuitable both in the present and in the future.

The CMCC-ESM2 climate model predicts that the species’ distribution will shrink significantly in the future. In the low-emission scenario (SSP1-2.6), habitat loss is expected to be around 48% by 2100, with roughly 40% of the existing range staying the same and about 12% of new regions being added. In the high-emission scenario (SSP5-8.5), on the other hand, losses are about 85% of the current range, persistence is only 1%, and gains stay at 5% (**Figure 5**; **Table 1**). The HadGEM3-GC31-LL climate model predicts a better outcome under SSP1-2.6, with habitat loss dropping to around 17% by 2100 and 32% of present habitats remaining. Gains will also grow to more than 50% of additional eligible regions. But things change a lot under SSP5-8.5: over 69% of habitats are lost, while 2% stay the same, and gains are limited to 8% (**Figure 6**; **Table 1**). The MIROC6 climate model predicts that under SSP1-2.6, the species will lose about 28% of its existing habitat by 2100. However, 55% of suitable places will still be there, and 17% of new habitat may be gained. Under SSP5-8.5, habitat loss grows worse, with around 69% of the present range lost, barely 1% left, and gains staying around 12% (**Figure 7**; **Table 1**).

**Table 1:** Range size change in *Cinclidotus bistratosus* under different years and scenarios.

Years-scenarios	Lost (%)	Abs (%)	Pres (%)	Gain (%)
CMCC-ESM2 climate model				
2021-2040 SSP1-2.6	50.41	97.29	1.32	1.63
2021-2040 SSP5-8.5	84.65	97.21	0.41	4.63
2041-2060 SSP1-2.6	49.94	97.15	1.34	6.95
2041-2060 SSP5-8.5	49.72	97.07	1.34	9.72
2061-2080 SSP1-2.6	40.25	96.75	1.59	21.89
2061-2080 SSP5-8.5	84.84	97.21	0.40	4.42
2081-2100 SSP1-2.6	47.76	97.01	1.39	12.22
2081-2100 SSP5-8.5	84.65	97.21	0.41	4.63
Hadgem3-GC31-LL climate model				
2021-2040 SSP1-2.6	30.51	96.54	1.85	29.74
2021-2040 SSP5-8.5	68.50	97.18	0.84	5.79
2041-2060 SSP1-2.6	27.12	96.85	1.94	18.04
2041-2060 SSP5-8.5	37.48	96.93	1.67	15.06
2061-2080 SSP1-2.6	19.94	96.04	2.14	48.54
2061-2080 SSP5-8.5	61.63	97.11	01.02	8.35
2081-2100 SSP1-2.6	16.63	95.95	2.22	51.85
2081-2100 SSP5-8.5	68.50	97.18	0.84	5.79
MIROC6 climate model				
2021-2040 SSP1-2.6	11.01	97.69	1.81	13.90
2021-2040 SSP5-8.5	69.47	97.81	0.62	7.56
2041-2060 SSP1-2.6	17.21	97.80	1.68	8.15
2041-2060 SSP5-8.5	33.83	97.84	1.34	6.39
2061-2080 SSP1-2.6	25.26	97.68	1.52	14.15
2061-2080 SSP5-8.5	57.59	97.71	0.86	12.49
2081-2100 SSP1-2.6	27.56	97.62	1.47	16.98
2081-2100 SSP5-8.5	69.47	97.81	0.62	7.56

## 4 Discussion

### 4.1 Current and future distributions of *C. bistratosus*

The potential distribution patterns of *C. bistratosus*, an endemic moss species for Türkiye, were compared under current and future climate scenarios. The CCCM-ESM2 climate model did not reflect a significant change in the current known distribution of the target species under the SSP1-2.6 climate scenario between the periods 2021-2100. In contrast, under the SSP5-8.5 scenario, the same model indicated a significant loss of habitat in the current distribution of the species during the time periods 2021-2040, 2061-2080 and 2081-2100. The HadGEM3-GC31-LL model shows that the simulated distribution range for *C. bistratosus* remains relatively stable for the SSP1-2.6 climate scenario. The model suggests that there will not be the same positive trend according to the SSP5-8.5 scenario. In the SSP5-8.5 scenario, the model signaled that there may be a limited habitat only in the southwestern part of the country, especially in the 2081-2100-time period. Both models (CCCM-ESM2 and HadGEM3-GC31-LL) depicted a relatively unfavorable scenario for the target species’ distribution areas according to the SSP5-8.5 climate scenario. It is assumed that this could be the possible effects of a temperature increase of 4.4°C according to the SSP5-8.5 scenario. Considering the habitat characteristics of the populations of the species in the available literature information (Kürschner and Erdağ, 2021), a direct response to water availability is also likely. It is evident that a considerable escalation in temperature and a decline in precipitation patterns may precipitate a diminution in available water resources and a contraction in the habitat of this hygrophytic species (Eissa and Zaki, 2011; Tokuşlu, 2022). Gürlek et al. (2024), in their study on predicting the threat status of mosses using different models and based on functional traits, correlated capsule and seta length with the future threat status of the species. Accordingly, they concluded that species with short capsule and/or seta length are more likely to be threatened. Similarly, they reported that the shorter the stem length, the higher the risk of a species being threatened. The authors also concluded that when the number of different substrates that a species can occupy is limited in terms of ecophysiologically relevant traits, the species is more likely to be threatened. Considering all these, the fact that our endemic moss species has very short setae, a stem length in the range of 2-5 cm, and a limited habitat preference, especially on submerged rocks, supports the hypotheses mentioned above.

The MIROC6 model simulated a wider habitat for *C. bistratosus* than the previous two models under both the SSP1-2.6 and SSP5-8.5 scenarios, suggesting that the species may exhibit a range of climate adaptation to high temperatures. *C. bistratosus* grows on rocks and/or submerged rocks. Preferred locations are wet rocks and cliffs that temporarily dry out and are exposed to high radiation when the water recedes in summer (Erdağ and Kürschner, 2011). The hygrophytic moss has some anatomical features for xerophytic living conditions. The presence of laminal papillose cells, strongly thickened of leaf margins, bistratose of the leaf lamina, very short seta and immersed capsules can be considered as a xeromorphic adaptation (Kürschner and Lübenau-Nestle, 2000). Vitt et al. (2014) pointed out that species with papillae usually have different cells in the upper part of the leaf than those in the lower part, noting that the cells in the upper part are photosynthetic with abundant chloroplasts and papillae, while the cells in the lower part of the leaf lack chloroplasts and papillae, but are larger, thinner-walled and transparent. The authors suggest that in this case the cells probably have a greater ability to retain water. They also explained that the thickening of the leaf surface could reduce evaporation and that this could be a way to prolong the time the cells can be active. In consequence, it can be posited that the potential for the species simulated by the MIROC6 model to exhibit a more extensive distribution area may be associated with its capacity for climatic adaptation.

The findings of this study indicate that BIO17 (precipitation of the driest quarter), BIO18 (precipitation of the warmest quarter) and BIO19 (precipitation of the coldest quarter) are the predominant variables influencing the potential distribution of *C. bistratosus*. A review of previous studies on rare and interesting moss species (Číhal et al., 2017; Spitale and Mair, 2017; Abubakar et al., 2024) and moss species with a narrow distribution range (Wu et al., 2023) has been conducted. In the context of *Didymodon validus*, which is distributed in China, studies have identified elevation and mean temperature in the wettest quarter as key factors influencing its distribution patterns (Wu et al., 2023). In the distribution modelling of rare and interesting species of the *Orthotrichum* genus in Tajikistan and Kyrgyzstan, the minimum temperature in the coldest month was found to act as a limiting factor for nearly every species (Číhal et al., 2017). In the study undertaken to ascertain the distribution of *Buxbaumia viridis* in northern Italy, two climatic variables (northness and rainfall) and two habitat-related variables (canopy closure and necromass) were identified as significantly determining factors (Spitale and Mair, 2017). A study examining the distribution of the rare and red-listed halophytic moss species *Entosthodon hungaricus* in Serbia under various climate change scenarios revealed that the rainfall of the driest month, rainfall seasonality, and average daily temperature range are among the most influential factors affecting the species’ development, as related to climatic characteristics (Abubakar et al., 2024).

### 4.2 Range changes under future climatic projections

Both SSP1-2.6 and SSP5-8.5 scenarios show notable temporal and regional shifts in species distribution, according to results of CMCC-ESM2. While there is some slight range extension in some eastern regions, habitat degradation is more noticeable in the western and southern regions. Compared to SSP1-2.6, the SSP5-8.5 scenario predicts more severe habitat loss, suggesting that range reductions are a direct result of greater emissions. Moderate habitat loss occurs between 2021 and 2040, while more substantial contractions occur between 2041 and 2060. By 2081-2100, few regions exhibit range expansion, and there is significant habitat loss, especially under SSP5-8.5. Net habitat loss is predicted for the species, with the largest decline taking place at the end of the century (**Figure 5**). In their study, Wysocki et al. (2024) concentrated on *Dicranum viride*, a moss species that is of conservation priority, and its reliance on specific phorophytes (host trees). The authors employed a range of SDM techniques and modelled the distribution of the phenomenon in question using climate-only variables. Furthermore, the authors developed a model to represent the distribution of the predominant phototroph species and incorporated this data into the *D. viride* SDM, along with data on climate. Considering each of the two SSP scenarios (SSP1-2.6 and SSP5-8.5) in their study, the less range contraction for *D. viride* is shown under SSP1-2.6. However, for the SSP5-8.5 scenario, range construction will be much more extensive. Our results are consistent with those of *D. viride*.

Under both SSP1-2.6 and SSP5-8.5 scenarios, the Hadgem3-GC31-LL climate model projections show significant changes in the distribution of the species under study. While there are noticeable localized contractions in the western and southwestern regions, the species exhibits a slight expansion of its distribution in certain northern and eastern regions during the 2021–2040 period. The years 2041–2060 show growing habitat loss as climate change intensifies, with contractions growing more widespread, especially in the range’s western region. With only a few isolated areas exhibiting the potential for persistence or increase, substantial habitat degradation predominates under SSP5-8.5 by 2081-2100 (**Figure 6**). Wang et al. (2025) showed that the total suitable habitat area of *Oryza sativa* tended to decrease under the scenario SSP2-4.5. The authors have stated that future increases in global temperatures, more frequent extreme weather events, and the expected intensification of human activities will cause the suitable distribution of *O. sativa* to continue to narrow. Similarly, it is expected that the habitat loss for *C. bistratosus* in Türkiye under future climate scenarios, especially under SSP5-8.5 according to the model Hadgem3-GC31-LL. The current locations of our target species, *C. bistratosus*, indicate areas with intense tourism activities. This situation may also lead to changes in the ecological distribution of the species over time and a decrease in suitable habitats. Tourism activities (e.g., rafting) in and around the habitats of our target species and the negative impacts of businesses established in the valleys (Köprülü and Dim) on the species’ habitat may pose a threat in the future. Zhang et al. (2018) found that changes in climate and land use will lead to a decrease in suitable habitats for *Paeonia delavayi* and *P. rockii* (peony) plants, that these species will be able to adapt to future climate conditions to a large extent, but that a significant portion of currently suitable habitats may disappear due to changes in land use and human activities for economic purposes. As also noted by Zhang et al. (2018), the reduction of suitable habitats due to land use for economic purposes supports our view.

In the climate model MIROC6, the species shows mild range alterations under SSP1-2.6, with reductions remaining mostly small and localized and expansions mostly taking place in northern and highland regions. But according to SSP5-8.5, habitat loss gets worse with time, with the worst contractions taking place in the second half of the twenty-first century (2061–2100). Significant areas of the species’ existing distribution, especially in western and southern Türkiye, are predicted to become unsuitable by 2081–2100 as a result of changing precipitation patterns and rising temperatures. An accelerating drop in population connection and even local extirpations are suggested by the rising rate of habitat loss under SSP5-8.5 (**Figure 7**). It is not surprising that the suitable habitat for *C. bistratosus*, which is adapted to aquatic areas, will decrease in the future and that expansions will mostly occur in northern and high-altitude regions, especially according to SSP5-8.5 (by 3.3°C to 5.7°C under the very high GHG emissions scenario), in the climate model MIROC6. Glime (2011) noted that factors associated with high temperatures have the capacity to alter the distribution of mosses and that the correlation between moss abundance and temperature in streams is typically negative. The author also emphasized the possibility that some aquatic systems exhibit more variable temperatures. Vanderpoorten et al. (1999) discovered that there was a negative correlation between the abundance of *Hygroamblystegium tenax*, *Chiloscyphus pallescens*, and *Pellia endiviifolia*, and the increase in the standard deviation of temperature. Furthermore, Vanderpoorten et al. (1999) found that *Cinclidotus danubicus* was not present in streams exhibiting a standard deviation of less than 4°C. Glime (2011) has mentioned that, along with climate change, many mosses will spread to higher latitudes and altitudes. The author identifies factors that will drive mosses toward higher latitudes and elevations, including higher respiration rates, reduced photosynthesis rates, lower available CO_2_, changing flow rates, increased desiccation events, and changing nutrient availability. Our simulation results indicate that expansions at the distribution points of our target species will occur at higher points and that habitat losses may occur with increasing temperatures, which supports the information provided above.

## 5 Conclusions

The distribution pattern of *C. bistratosus* is primarily influenced by three key environmental variables: precipitation during the driest, warmest and coldest quarters. Overall, while there are climatic and spatial changes that will be experienced over a long period of time, it is under the SSP5-8.5 climate scenario that we see radical habitat contraction and change. In the context of future climate warming, the suitable habitat centre of *C. bistratosus* may move towards northern and high-altitude regions under the SSP5-8.5 climate scenario. However, it will undergo a partial withdrawal from its current Mediterranean distribution range. Its potential distribution range is predicted to remain confined to Türkiye.

Given that *C. bistratosus* is endemic to Türkiye, the constructed simulations enable the search for new suitable microhabitats and populations in the country. In the first place, the natural habitats where the species is distributed should be better protected and managed. Concerted efforts are imperative to establish effective monitoring and conservation strategies to prevent any decline in population numbers in natural habitats. Here, the importance of protecting riparian habitats and implementing water management strategies to mitigate climate-induced habitat loss becomes evident. For further research and conservation planning, the focus should be on monitoring known populations and identifying potential future habitats.

Although ecological niche modeling (ENM) is a powerful tool, there are various limitations to be considered in the interpretation of the findings. Since the model is based on statistical correlations between species records and environmental variables, it does not directly represent biological mechanisms (such as interreligious competition, hunter relations). In addition, it does not contain other important factors that may affect the spread of the species such as land use, soil types and geological properties. Despite these limitations, the results of our study show that both high emissions (SSP5-8.5) under different climatic models will lead to serious loss of habitat. Likewise, in the scenario with low emissions (SSP1-2.6), it was observed that habitat losses decreased significantly and the potential of the species to gain new habitat increased. These consistent findings support the scientific validity of the general trends offered by the model. In this way, our model provides a valuable starting point for the determination of protection strategies and the determination of sensitive areas against the potential effects of climate change.

## Data Availability

The raw data supporting the conclusions of this article will be made available by the authors, without undue reservation.
